# Publisher Correction: Age and life expectancy clocks based on machine learning analysis of mouse frailty

**DOI:** 10.1038/s41467-020-19046-8

**Published:** 2020-10-08

**Authors:** Michael B. Schultz, Alice E. Kane, Sarah J. Mitchell, Michael R. MacArthur, Elisa Warner, David S. Vogel, James R. Mitchell, Susan E. Howlett, Michael S. Bonkowski, David A. Sinclair

**Affiliations:** 1 Blavatnik Institute, Department of Genetics, Paul F. Glenn Center for Biology of Aging Research at Harvard Medical School, Boston, MA USA; 2 Charles Perkins Centre, The University of Sydney, Sydney, NSW Australia; 3 Department of Genetics and Complex Diseases, Harvard T.H. Chan School of Public Health, Boston, MA USA; 4 Department of Computational Medicine & Bioinformatics, University of Michigan, Ann Arbor, MI USA; 5 Voloridge Investment Management, LLC and VoLo Foundation, Jupiter, FL USA; 6 Departments of Pharmacology and Medicine (Geriatric Medicine), Dalhousie University, Halifax, NS Canada; 7 Department of Dermatology, The Feinberg School of Medicine, Northwestern University, Chicago, IL USA; 8 Department of Pharmacology, School of Medical Sciences, The University of New South Wales, Sydney, NSW Australia

**Keywords:** Computational models, Machine learning, Ageing

Correction to: *Nature Communications* 10.1038/s41467-020-18446-0, published online 15 September 2020.

The original version of this Article contained an error in the Competing interest statement. It was incorrectly stated that D.S.V. sits on the board of Life Biosciences and Iduna. The correct declaration is D.A.S sits on the board of both companies. This has now been corrected in both the PDF and HTML versions of the Article.

