# Supplementary material for: Age and life expectancy clocks based on machine learning analysis of mouse frailty
Source: Nat Commun. 2020 Sep 15;11:4618. doi: 10.1038/s41467-020-18446-0 (PMC7492249; doi:10.1038/s41467-020-18446-0)
Supplement: Supplementary file 1 — Supplementary Information [file 41467_2020_18446_MOESM1_ESM.pdf]

Supplementary Information File

Age and life expectancy clocks based on machine learning analysis of mouse frailty

Schultz, Kane et al. 2020

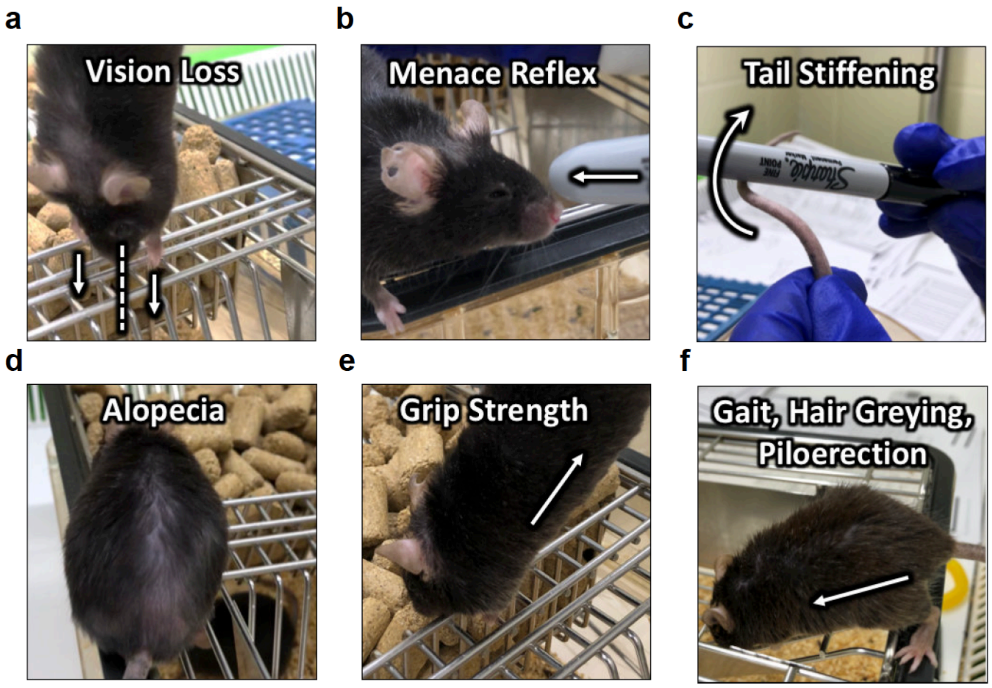

g

Integument				Notes	Ocular/Nasal				Notes
Alopecia	0	H	1		Cataracts	0	H	1	
Fur color loss	0	H	1		Corneal opacity	0	H	1	
Dermatitis	0	H	1		Eye discharge/swelling	0	H	1	
Loss of whiskers	0	H	1		Microphthalmia	0	H	1	
Coat condition	0	H	1		Vision loss	0	H	1	
**Physical/Musuloskeletal**					Menace reflex	0	H	1	
Tumors	0	H	1		Nasal discharge	0	H	1	
Distended abdomen	0	H	1		**Digestive/Urogenital**				
Kyphosis	0	H	1		Malocclusions	0	H	1	
Tail stiffening	0	H	1		Renal prolapse	0	H	1	
Gait disorders	0	H	1		Vaginal/uterine/ penile prolapse	0	H	1	
Tremor	0	H	1		Diarrhoea	0	H	1	
Grip strength	0	H	1		**Respiratory system**				
Body condition	0	H	1		Breathing rate/depth	0	H	1	
**Vestibulocochlear/Auditory**					**Discomfort**				
Vestibular disturb.	0	H	1		Mouse grimace	0	H	1	
Hearing loss	0	H	1		Piloerection	0	H	1	

Supplementary Figure 1. Frailty Index (FI) items. (a-f) Examples of deficits measured with the non-invasive mouse clinical FI assessment[14]: (a) vision loss as assessed by the point at which the mouse reaches for the cagetop when lowered; (b) menace reflex as assessed by aggressive response to an object out towards the face; (c) tail stiffening as assessed by the ability of the tail to curl around an object; (d) alopecia or hair loss; (e) grip strength assessed by the force with which the mouse grips the cage top; (f) gait as assessed by a mouse walking on an incline; hair greying and piloerection or the amount the fur stand on end. For more detail see: http://frailtyclocks.sinclairlab.org/ (g) Scoresheet for automated data entry of FI item scoring, modified from original paper (available at https://github.com/SinclairLab/frailty).

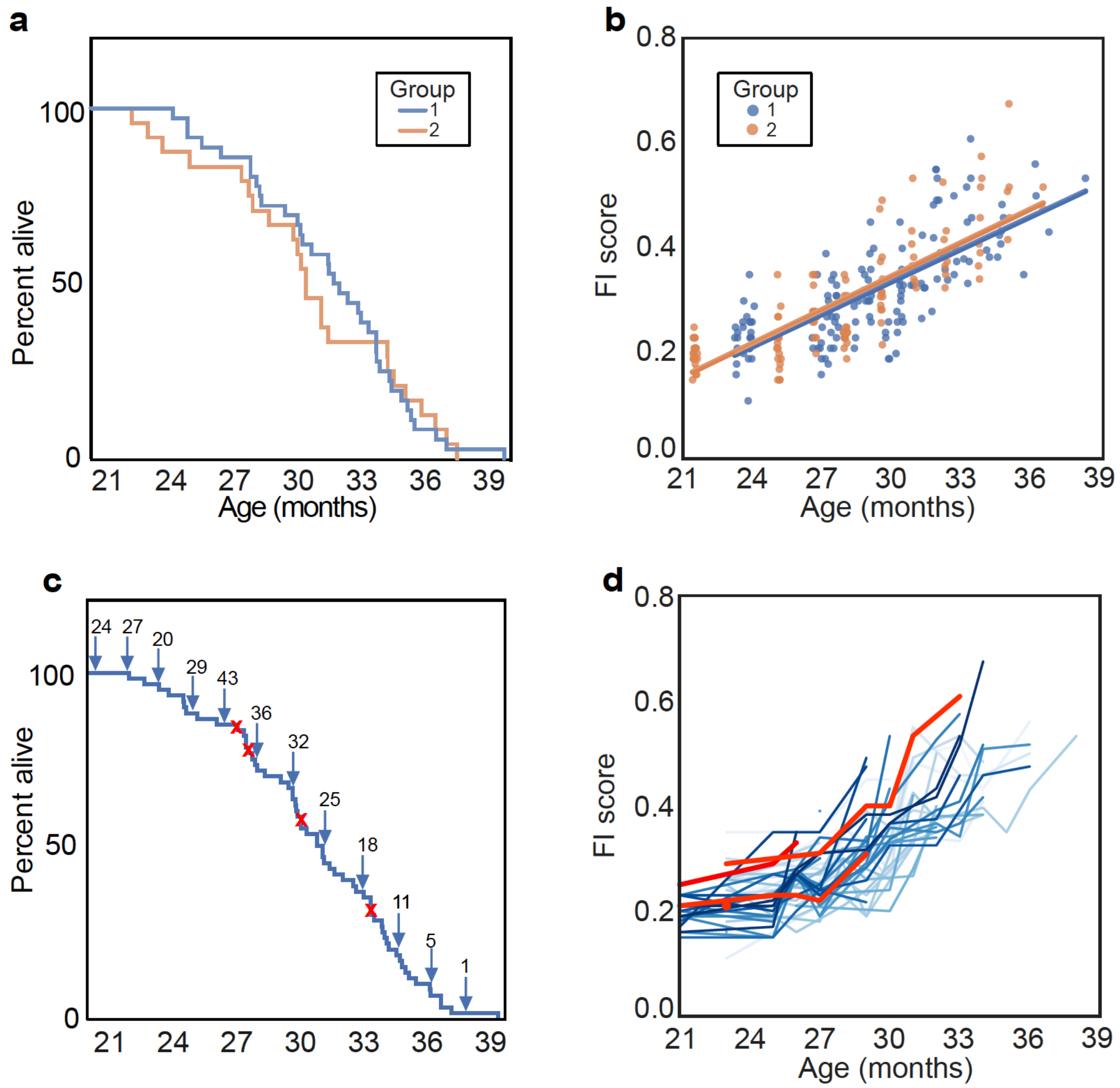

Supplementary Figure 2. Mortality curves and frailty index for mice cohorts. (a) Kaplen-Meier curves for male C57BL/6 mice included in the current study. One cohort of mice (blue, n=36) was injected with AAV vectors containing GFP as a control group for a separate longevity experiment, while the second cohort (orange, n=24) was untreated. (b) All FI scores from 21 months of age for mice included in the current study. (c) Based on Figure 1A. Kaplan-Meier survival curve for all mice included in the current study, showing n value at each assessment point, and which mice were euthanized (n=4, red crosses). (d) Based on Figure 1C. FI score trajectories for each individual mouse included in the current study, with mice that were euthanized shown in red.

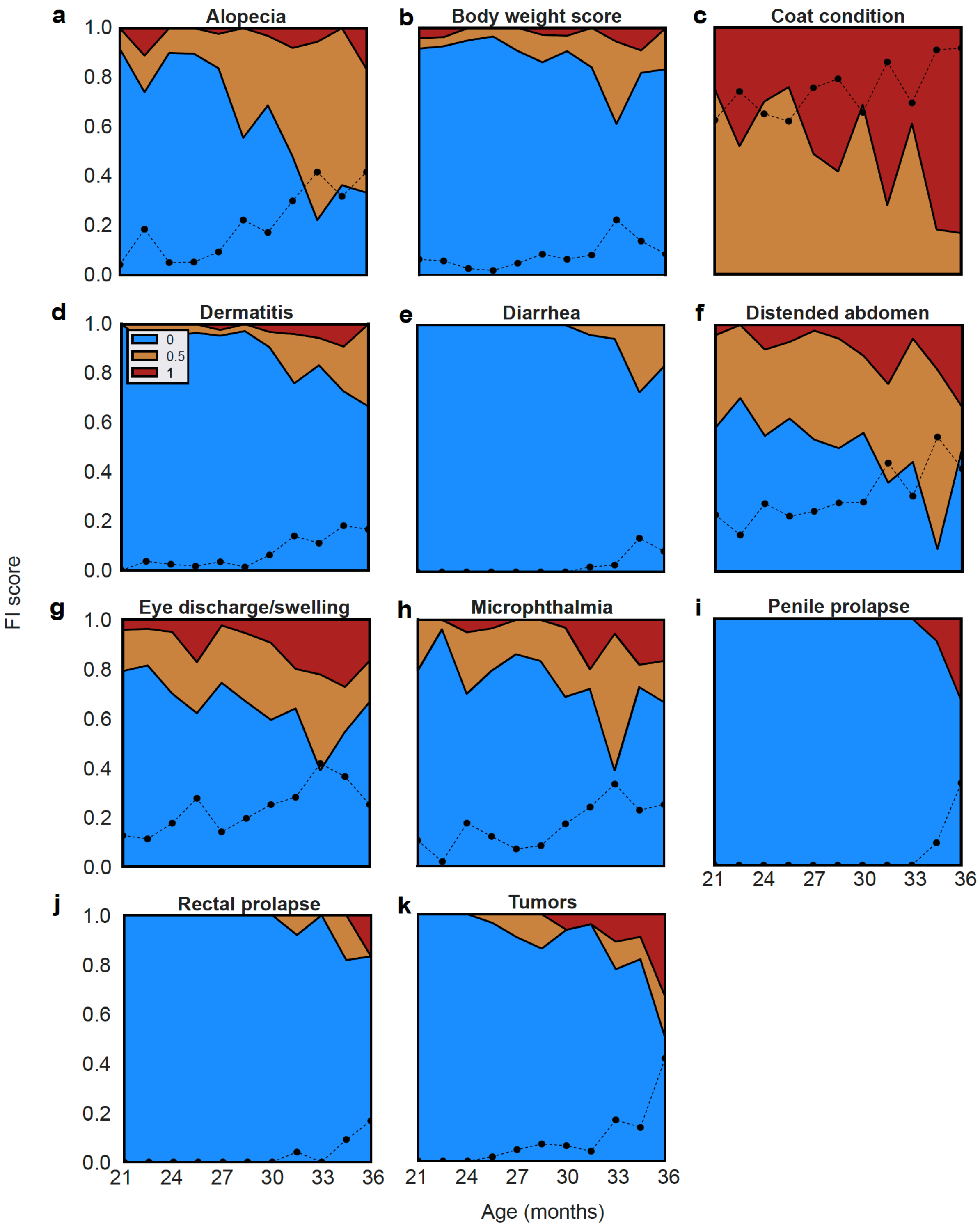

Supplementary Figure 3. Individual FI items vary in their correlation with age. Mean scores across all mice (black line) for individual items of the frailty index from 21 to 36 months of age that had any positive correclation with age. Colors indicate proportion of mice at each age with each score (0, blue; 0.5, orange, 1, red).

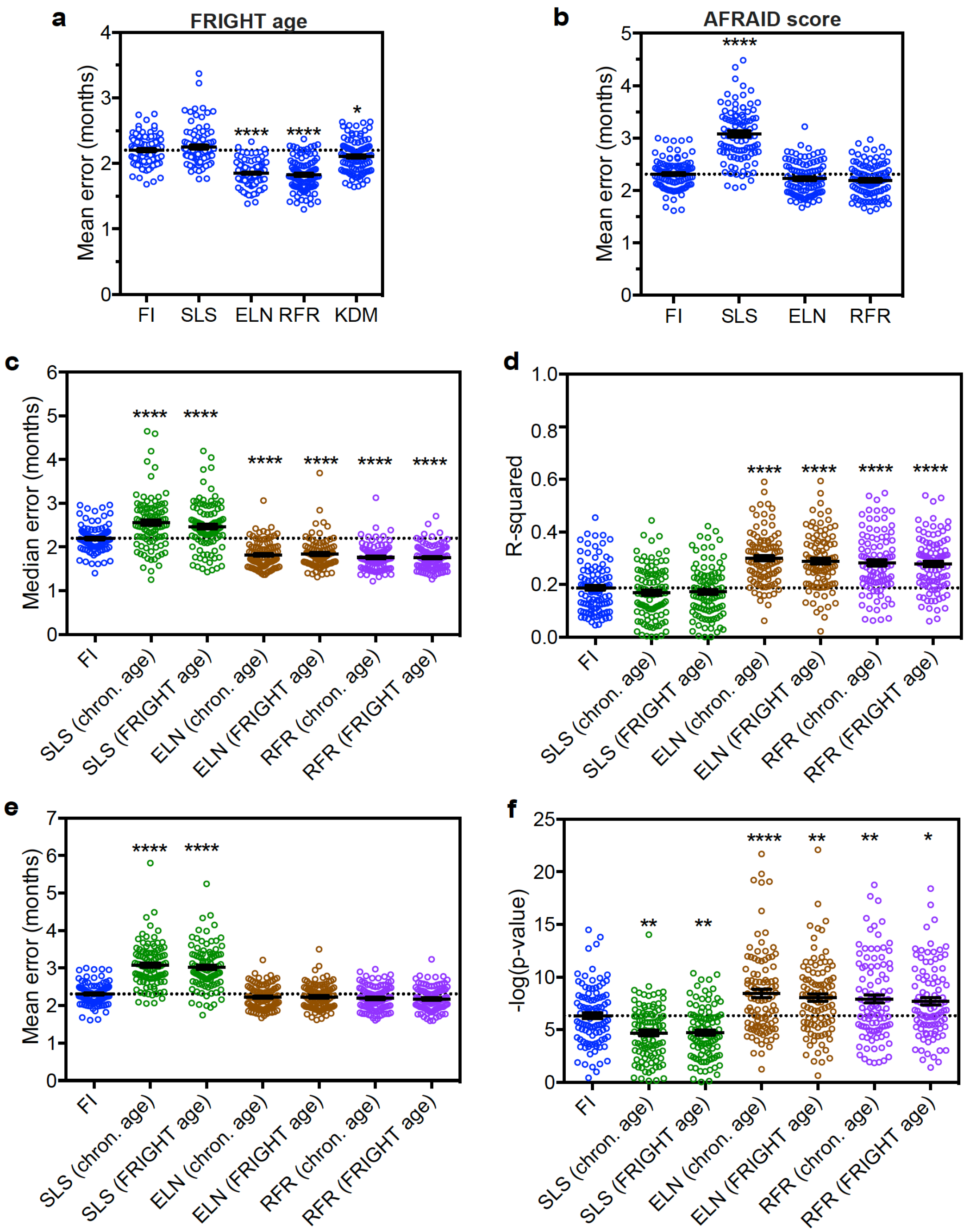

Supplementary Figure 4. Mean errors for multivariate regressions to predict age and lifespan, and bootstrapping results for AFRAID model including FRIGHT age as a variable. (a) Mean error for univariate regression of frailty index (FI) score, and multivariate regressions of the individual FI items using either simple least squares (SLS), elastic net (ELN), the Klemera-Doubal method (KDM), or random forest regression (RFR) for chronological age in the mouse training set. (b) Mean error for univariate regression of FI score, and multivariate regressions of the individual FI items using either SLS, ELN or RFR for life expectancy in the mouse training set. (c-f) Median error,

mean error, r^2 values and p-values for univariate regression of FI score, and multivariate regressions of the individual FI items using either SLS, ELN or RFR for life expectancy in the mouse training set. Each model includes either chronological (chron.) age or FRIGHT age as a variable. All models were tested with bootstrapping with replacement repeated 100 times, and each bootstrapping incidence is plotted as a separate point. **** indicates p-value <0.0001, ** indicates p-value <0.01 and * indicates p-value <0.05 compared to FI model with one-way ANOVA. Error bars represent standard error of the mean.

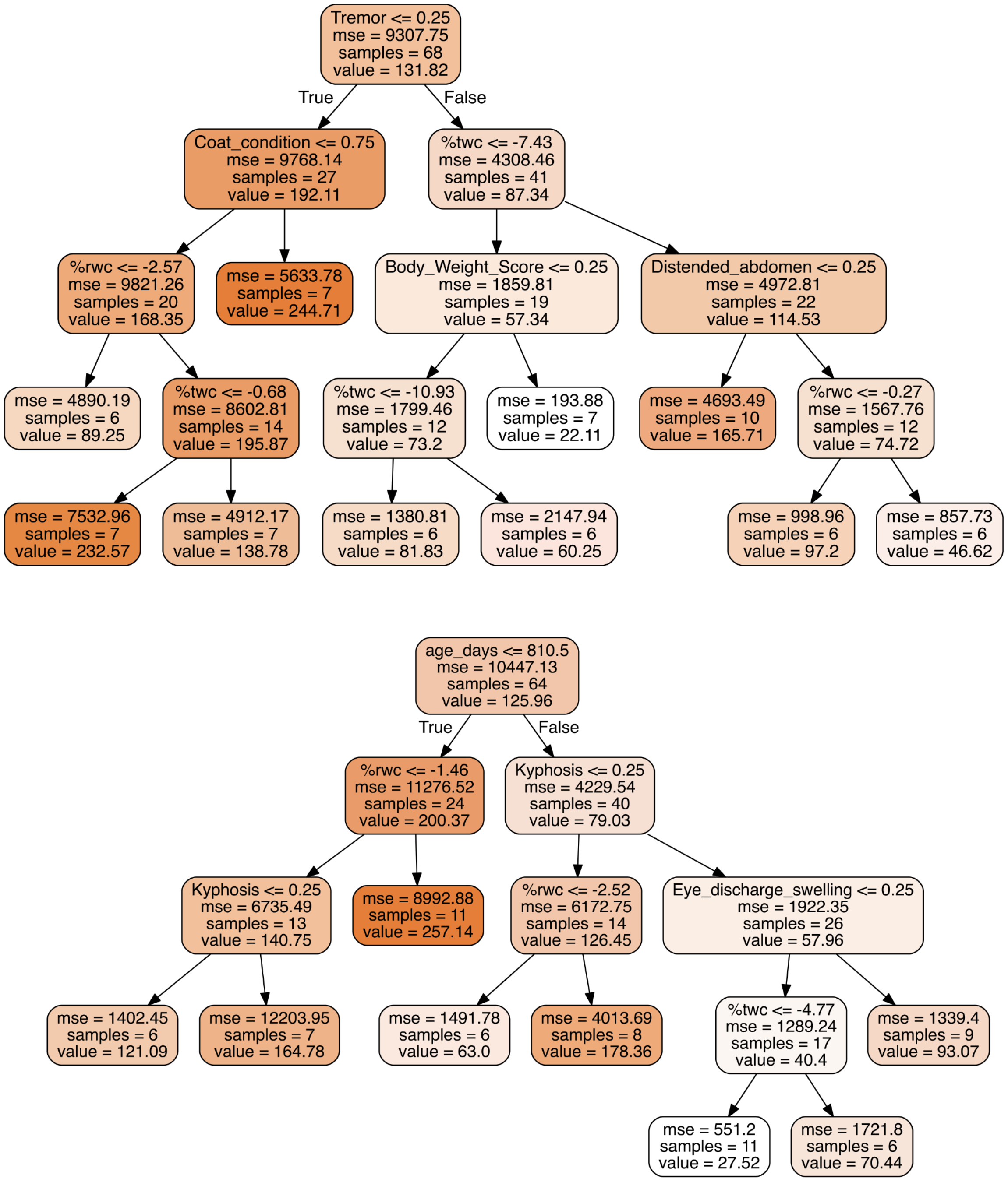

Supplementary Figure 5. Example tree from random forest analysis. An illustration of two decision trees, out of the one thousand that comprise the AFRAID clock measure.

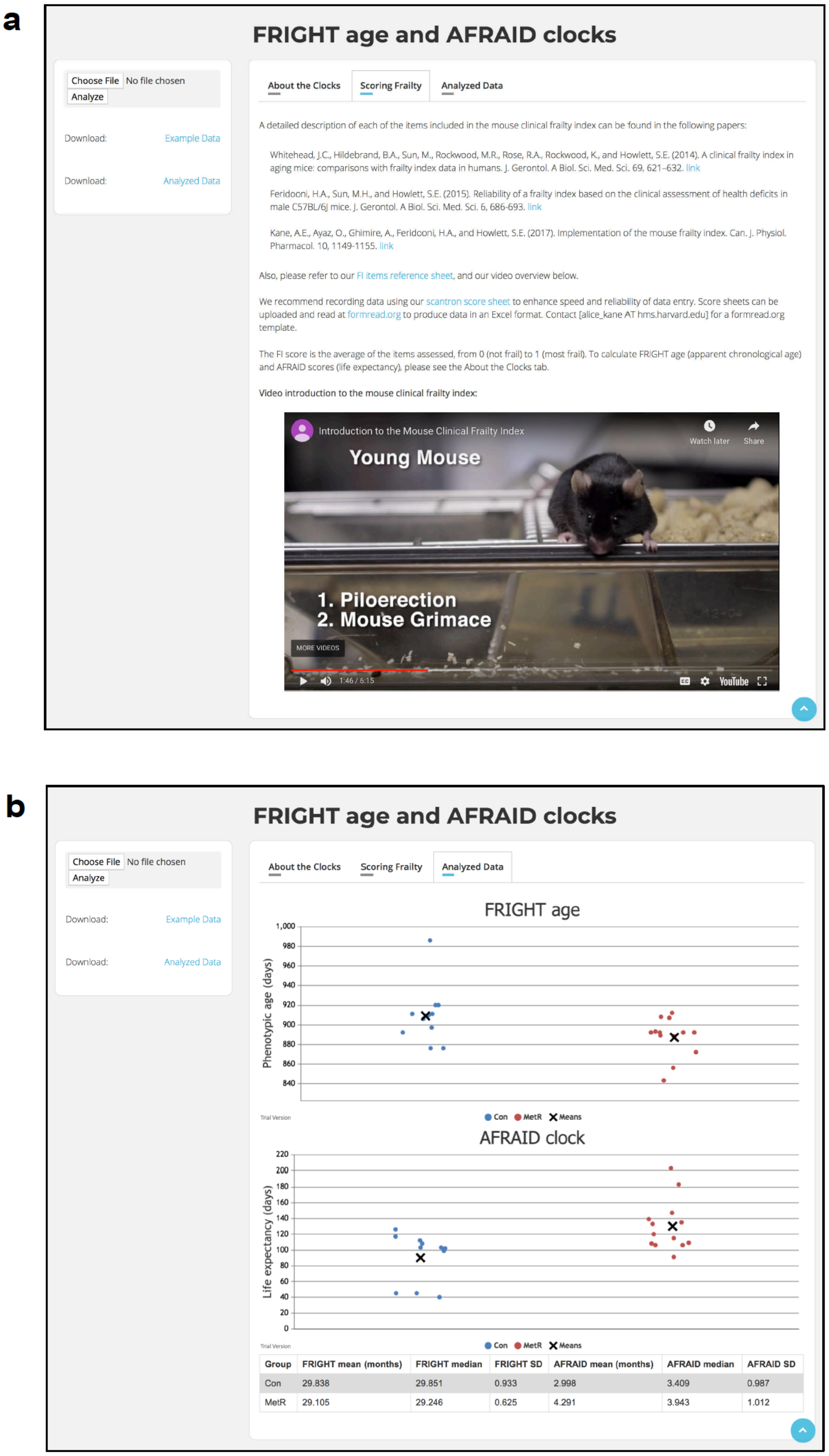

Supplementary Figure 6. Website for implementation of frailty clocks. (a) A screenshot of frailtyclocks.sinclairlab.org with resources describing how to score the mouse clinical frailty index, including a step-by-step video guide. (b) A screenshot showing data analyzed using the

website. Using the buttons in the box on the left, raw frailty scores are uploaded, FRIGHT and AFRAID scores are calculated, and analyzed data can be downloaded. The larger box on the right displays graphs and tables summarizing the data.
